# The efficacy of VEGFR TKI therapy after progression on immune combination therapy in metastatic renal cell carcinoma

**DOI:** 10.1038/s41416-018-0104-z

**Published:** 2018-05-24

**Authors:** Pedro Coelho Barata, Alfonso Gomez De Liano, Prateek Mendiratta, Valerie Crolley, Bernadett Szabados, Laura Morrison, Laura Wood, Kimberly Allman, Allison Tyler, Allison Martin, Timothy Gilligan, Petros Grivas, Moshe Ornstein, Jorge A. Garcia, Thomas Powles, Brian I. Rini

**Affiliations:** 1 Department of Hematology and Medical Oncology, Taussig Cancer Institute, Cleveland Clinic, Cleveland, OH USA; 2 Barts Cancer Institute, Barts and The London School of Medicine and Dentistry, Queen Mary of London, London, UK

**Keywords:** Renal cancer, Cancer

## Abstract

**BACKGROUND:**

The outcome of patients who progress on front-line immune-based combination regimens (IC) including immune checkpoint inhibitors (CPI) and receive subsequent systemic therapy is unknown.

**Methods:**

Retrospective analysis of consecutive patients with clear-cell mRCC who progressed on one of seven clinical trials investigating an IC and received ≥1 line of subsequent VEGFR TKI therapy.

**Results:**

Thirty-three patients [median age 57 (37–77), 85% male, 73% ECOG 0] were included. For evaluable patients (*N* = 28), the best response to first subsequent therapy was 29% partial response, 54% stable disease, and 18% progressive disease. The median PFS (mPFS) for first subsequent therapy was 6.4 months (95% CI, 4.4–8.4); no difference in mPFS by prior type of IC (VEGFR TKI-CPI vs. CPI-CPI) was noted (*p* = 0.310). Significant AEs were observed in 30% of patients, more frequently transaminitis (9%).

**Conclusions:**

VEGFR TKIs have clinical activity in mRCC refractory to IC therapy, possibly impacted by the mechanism of prior combination therapy.

## Introduction

Medical treatment for metastatic renal cell carcinoma (mRCC) has expanded considerably from a nonspecific immune approach to targeted therapies against vascular endothelial growth factor receptor (VEGFR) and mammalian target of rapamycin (mTOR).^1–5^ More recently, the survival benefit and subsequent approval of the immune checkpoint inhibitor (CPI) nivolumab, for patients with refractory mRCC has ushered in a new era of research into potential combination approaches to improve outcomes and to potentially overcome resistance.^6^ Preclinical data suggesting synergy between angiogenic inhibition and the combination of anti-cytotoxic T lymphocyte-associated antigen-4 with CPIs has led to the development of immune-oncology-based combination (IC) regimens.^7, 8^ Early-phase trials with different IC regimens resulted in promising clinical activity with high response rates and manageable tolerability in heavily treated mRCC patients.^9–11^ The combination of nivolumab and iplimumab resulted in higher overall response rate and prolonged overall survival (OS) compared to sunitinib in intermediate and poor-risk mRCC patients (Checkmate-214) and other phase III studies testing several ICs are ongoing thus, the front-line treatment of mRCC is likely to change.^12^

Unfortunately, most patients eventually progress and require additional treatment after an IC regimen. The clinical outcome of patients who receive subsequent systemic therapy is undetermined. To assess the safety and efficacy of subsequent treatments in the post-immunotherapy setting, we conducted a retrospective review of consecutive mRCC patients from two academic centres who had received a prior IC and were subsequently treated with a targeted agent.

## Methods

This was a retrospective, multi-institutional study that included patients with clear-cell mRCC enroled in one of the seven clinical trials investigating a combination regimen including a CPI (NCT02420821, NCT01984242, NCT01472081, NCT02231749, NCT02853331, NCT02493751, NCT02684006). All patients were required to have disease progression at the discretion of the treating physician and were subsequently treated with at least one VEGFR-TKI. Patients who remained on all or part of the IC regimen after coming off trial were excluded. Patients were grouped into those who had received a combination of CPI and VEGFR-TKI and those who had received a combination of two CPIs.

### Study population

Eighty-nine mRCC patients enroled in one of the seven clinical trials investigating an IC at two institutions (Taussig Cancer Center Cleveland Clinic, Cleveland; Barts Cancer Institute, London) were retrospectively collected, in compliance with IRB guidelines. Electronic medical records were reviewed for baseline characteristics. Patients were categorised into three risk categories: favourable-risk, intermediate-risk and poor-risk according to The International Metastatic Renal Cell Carcinoma Database Consortium (IMDC) risk criteria.^13^ Outcome measures were also retrieved from chart reviews including progression-free survival (PFS), investigator-assessed overall response rate (ORR) according to RECIST v1.1^14^ and adverse events (AEs) using CTCAE v4.0.^15^

### Statistical analysis

The primary objective of this study was to characterise ORR and PFS of mRCC patients treated with at least one subsequent therapy after progression on IC. The data reported here (including OS) was limited to protect the ongoing phase 3 trials testing ICs that have not yet been presented. PFS was defined as the time period between first subsequent treatment initiation and drug discontinuation due to progression, death, or last follow-up. Those who were still on treatment and those who discontinued therapy without disease progression were censored at the date of last evaluation. Distributions of PFS were estimated using Kaplan–Meier methodology. The statistical analysis was performed using SPSS version 23 and *p* < 0.05 was considered statistically significant.

## Results

### Baseline characteristics

Between February 2015 and September 2017, 89 patients with mRCC enroled on an IC protocol. Among the 41 patients who progressed, 8 patients were excluded: five patients remained on axitinib at an escalated dose after coming off trial with a combination regimen that included axitinib; one patient received nivolumab as first subsequent therapy after IC and two patients died before starting subsequent therapy. The final cohort included 33 patients who progressed on IC protocol. Twenty-four patients were on combo at time of PD, while four patients were on single-agent VEGFR TKI, three on CPI alone and two other had both IC agents on hold due to toxicity.

Patient characteristics are summarised in Table 1. Twenty-two patients received a VEGFR-TKI in combination with a CPI, and 11 patients had a combination of two CPIs. Thirty-two patients received IC in the front-line setting while one patient was treated with ipilumumab plus nivolumab after prior treatment with front-line sunitinib.

**Table 1:** Baseline characteristics prior to first subsequent therapy

Characteristics	%
Median age	57 (37–77)
Gender	Male: 85%
	Female: 15%
PS	0: 73%
	1: 27%
IMDC risk group	Favourable: 27%
	Intermediate: 52%
	Poor: 21%
Location of metastases	Lung: 76%
	Lymph nodes: 52%
	Bone: 30%
	Liver: 21%
	Locoregional: 21%
Prior nephrectomy	64%
IC regimen	Atezolizumab/bevacizumab: 64%
	Ipilimumab/nivolumab: 33%
	Axitinib/avelumab: 3%
Number of subsequent systemic therapies	1: 100%
	2: 36%
	≥3: 15%
First subsequent systemic therapy (*n* = 31)	Axitinib: 48%
	Cabozantinib 12%
	Pazopanib: 27%
	Sunitinib: 12%
Second subsequent systemic therapy (*n* = 12)	Axitinib: 25%
	Cabozantinib: 8%
	HIF inhibitor: 8%
	Lenvatinib/everolimus: 8%
	mTOR inhibitor: 8%
	Nivolumab: 8%
	Sorafenib: 8%
	Sunitinib: 8%
	Tivozanib: 8%
Third subsequent systemic therapy (*n* = 5)	Cabozantinib: 80%
	Everolimus: 20%

### Outcomes of subsequent treatment after IC

**Fig. 1:**
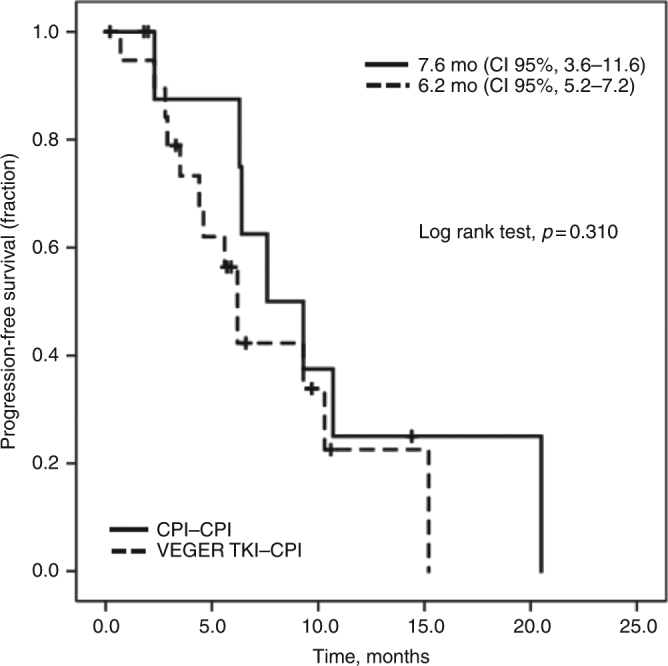
Progression-free survival (PFS) curve by type of IC. *mPFS* median progression-free survival, *mo* months, *CI* confidence interval, *CPI* checkpoint inhibitor, *IC* immune-based combination

The median follow-up time from the initiation of the first subsequent therapy was 13 months. All patients received one subsequent therapy (axitinib *n* = 16; pazopanib *n* = 9; sunitinib *n* = 4; cabozantinib *n* = 4) initiated after a median of 27 days (range, 0–2 years) after disease progression on the IC regimen. Twelve patients were treated with a second subsequent therapy and five patients were treated with ≥3 subsequent lines of treatment (Table 1). The median PFS and ORR for each first subsequent therapy after progression on ICs are summarised in Table 2. For patients with available response data (*n* = 28), the overall best response to first subsequent therapy were partial response (PR) in 8 (29%), stable disease (SD) in 15 (54%), and progressive disease (PD) in 5 (18%; Table 2). A higher proportion of RR was observed with pazopanib (43%) while patients who received sunitinib had no responses and 50% (2/4) had PD as best response. The ORR was 33% for patients previously treated with combination of two CPIs compared with 25% for CPI plus anti-VEGFR combination (*p* *=* 0.678). Median PFS for the first subsequent therapy was 6.4 months (95% CI, 4.4–8.4) with seven patients remaining on treatment. The median PFS for cabozantinib was not reached, while PFS for sunitinib was 2.9 months compared with 5.6 and 6.4 months for pazopanib and axitinib, respectively (Table 2). The median PFS for patients previously treated with a combination of anti-VEGFR plus CPI was 6.2 months (95% CI, 5.2–7.2) and 7.6 months (95% CI, 3.6–11.6) for patients treated with a combination of two CPIs (*p* = 0.310; Fig. 1). A total of 12 patients received a second subsequent therapy after IC, with a median PFS of 4.1 months (95% CI, 0–9.2).

**Table 2:** Progression-free survival (PFS) and best response (RECIST v1.1) to first subsequent treatment in evaluable patients

	Axitinib (*N* = 14)	Pazopanib (*N* = 7)	Cabozantinib (*N* = 3)	Sunitinib (*N* = 4)	Total cohort(*N* = 28)
Median PFS, months (CI 95%)	6.4^a^ (4.7–8.1)	5.6 (1.2–10.0)	NR	2.9 (0.0–7.6)	6.4^a^ (4.4–8.4)
Objective response rate (ORR)	29% (4)	43% (3)	33% (1)	0% (0)	29% (8)
Stable disease (SD)	64% (9)	29% (2)	66% (2)	50% (2)	54% (15)
Progressive disease (PD)	7% (1)	29% (2)	0% (0)	50% (2)	18% (5)

*NR* not reached

^a^One patient who received axitinib in the third-line setting was excluded

Any AE occurred in 73% of the patients treated with first subsequent therapy (Table 3). Overall, the most frequent AEs (any grade) were fatigue (36%), diarrhoea (30%) and mucositis (24%). Significant (G3/G4) AEs were reported in 10 patients and included LFTs elevation (*n* = 3), diarrhoea (*n* = 2), mucositis (*n* = 2), fatigue (*n* = 1), hypertension (*n* = 1), low platelet count (*n* = 1), and nephrotic syndrome (*n* = 1). One patient developed G4 AE (elevated LFTs) while on pazopanib. For patients with G3/4 AEs, the median time between the last IC and the first dose of VEGFR-TKI was 20 days (range, 0–64). Considering all patients with significant LFTs abnormalities (*n* = 3), two started pazopanib 1 day after stopping IC (ipilimumab/nivolumab and atezolizumab/bevacizumab), respectively, while one patient started axitinib 4 weeks after ipilimumab/nivolumab discontinuation. No association was found between time to first subsequent TKI and significant AEs (*p* = 0.288).

**Table 3:** Grade 2–4 adverse events of any cause during first subsequent TKI

Factor	Grade 2	Grade 3	Grade 4	Total
	*N* (%)			
Anorexia	2 (6)	0 (0)	0 (0)	2 (6)
Diarrhoea	5 (15)	2 (6)	0 (0)	7 (21)
Fatigue	6 (18)	1 (3)	0 (0)	7 (21)
Hand foot syndrome	1 (3)	0 (0)	0 (0)	1 (3)
Hypertension	2 (6)	1 (3)	0 (0)	3 (9)
Hypothyroidism	2 (6)	0 (0)	0 (0)	2 (6)
Mucositis	5 (15)	2 (6)	0 (0)	7 (21)
Nausea	2 (6)	0 (0)	0 (0)	2 (6)
Nephrotic syndrome	0 (0)	1 (3)	0 (0)	1 (3)
Vomiting	1 (3)	0 (0)	0 (0)	1 (3)
AST/ALT elevation	0 ()	2 (6)	1 (3)	3 (9)
Low platelets	0 (0)	1 (3)	0 (0)	1 (3)

## Discussion

This analysis evaluated the activity of subsequent treatments after failure of front-line IC regimens in mRCC, for which there is currently a lack of data due to the rapidly evolving field. The response and PFS observed with first subsequent therapy confirmed the activity of VEGFR TKIs in this setting and in line with the standard second line therapy after progression on TKI therapy.^16, 17^ Of note, a numerical advantage in objective response and PFS to subsequent was observed in patients who did not receive prior anti-VEGF agents, although not statistically significant. This is likely due to the reduced activity of anti-VEGF agents when given in sequence, although larger prospective data are required.

This work supports the continued sequencing of these agents in mRCC to maximise outcomes, although differences in clinical activity among different TKIs were not able to be discerned in this small, retrospective series and will require prospective investigation.

In addition, insight into potential toxicity was noted in this series including preliminary signals, such as LFT elevation. CPIs have a prolonged half-life, and thus a longer washout period is recommended to prevent interactions with subsequent therapies. As this is an area of significant risk, this transition period requires frequent laboratory and clinical assessment.

There are several limitations of this analysis, including the retrospective nature, a relatively small sample size, the diversity of TKIs in the post-IO space and short follow up. Importantly, only patients enroled in a clinical trial testing an IC were included, who are known to have favourable clinical characteristics and not necessarily fully representative of the mRCC population seen in clinical practice.^18, 19^ Nevertheless, these findings show clinical activity of anti-VEGF agents with an acceptable safety signal when given after immune-based combination therapy, supporting the sequencing of these agents as a standard of care. Validation of these findings in future clinical trials is warranted, and such trials are ongoing including the ongoing phase II studies assessing the clinical activity of pazopanib (NCT03200717) or an individualised schedule of axitinib (NCT02579811) after prior CPIs.
